# Children Learning About Secondhand Smoke (CLASS II): protocol of a pilot cluster randomised controlled trial

**DOI:** 10.1136/bmjopen-2015-008749

**Published:** 2015-08-25

**Authors:** Kamran Siddiqi, Rumana Huque, Cath Jackson, Steve Parrott, Omara Dogar, Sarwat Shah, Heather Thomson, Aziz Sheikh

**Affiliations:** 1 Department of Health Sciences, The University of York, York, UK; 2 ARK Foundation, Dhaka, Bangladesh; 3 Department of Public Health, Leeds City Council, Leeds, UK; 4 Centre for Medical Informatics, Usher Institute of Population Heath Sciences and Informatics, The University of Edinburgh, Edinburgh, UK

**Keywords:** second-hand smoking, passive smoking, children, school, tobacco

## Abstract

**Introduction:**

Exposure to secondhand smoke (SHS) increases children’s risk of acquiring chest and ear infections, tuberculosis, meningitis and asthma. Smoking bans in public places (where implemented) have significantly reduced adults’ exposure to SHS. However, for children, homes remain the most likely place for them to be exposed to SHS. Additional measures are therefore required to protect children from SHS. In a feasibility study in Dhaka, Bangladesh, we have shown that a school-based smoke-free intervention (SFI) was successful in encouraging children to negotiate and implement smoking restrictions in homes. We will now conduct a pilot trial to inform plans to undertake a cluster randomised controlled trial (RCT) investigating the effectiveness and cost-effectiveness of SFI in reducing children’s exposure to SHS.

**Methods and analysis:**

We plan to recruit 12 primary schools in Dhaka, Bangladesh. From these schools, we will recruit approximately 360 schoolchildren in year 5 (10–12 years old), that is, 30 per school. SFI consists of six interactive educational activities aimed at increasing pupils’ knowledge about SHS and related harms, motivating them to act, providing skills to negotiate with adults to persuade them not to smoke inside homes and helping families to ‘sign-up’ to a voluntary contract to make their homes smoke-free. Children in the control arm will receive the usual education. We will estimate: recruitment and attrition rates, acceptability, fidelity to SFI, effect size, intracluster correlation coefficient, cost of intervention and adverse events. Our primary outcome will consist of SHS exposure in children measured by salivary cotinine. Secondary outcomes will include respiratory symptoms, lung function tests, healthcare contacts, school attendance, smoking uptake, quality of life and academic performance.

**Ethics and dissemination:**

The trial has received ethics approval from the Research Governance Committee at the University of York. Findings will help us plan for the definitive trial.

**Trial registration number:**

ISRCTN68690577.

**Strengths and limitations of this study**

- A pilot to inform the parameters required for a pioneering trial to test the effectiveness and cost-effectiveness of a school-based behavioural change intervention aimed at children to negotiate and implement smoking restrictions in homes.
- A pilot that is rooted in a well-developed smoke-free intervention (SFI) that has shown promising results from the feasibility study in Dhaka, Bangladesh.
- The first study of secondhand smoke (SHS) exposure in children that will assess a variety of clinical, behavioural and school performance-related outcomes (in children), such as the frequency and severity of respiratory illnesses, healthcare contacts, school absenteeism, smoking uptake and improvements in their lung function, quality of life and academic performance.
- The only limitation is that the trial is not powered to show the effectiveness of the SFI, which can be attributed to its design being a pilot trial to inform plans of a bigger definitive cluster RCT in the future.

## Introduction

Secondhand smoke (SHS) contains an estimated 4000 toxic chemicals and is a serious health hazard to non-smokers. Every year, an estimated 600 000 people die due to SHS exposure.^1^ Exposure to SHS is particularly harmful to children's health and increases their risk of acquiring lower respiratory tract and middle ear infections,^2–4^ invasive meningococcal disease,^5^ tuberculosis^6^ ^7^ and incident cases, recurrent episodes and increased severity of asthma.^8^ Parental smoking is also associated with their children's admissions to hospitals.^3^ Children living in smoking households are at risk of poor general and functional health,^9^ lower academic performance,^10^ and a high smoking uptake in later life.^11^

Recognising SHS as a public health threat, most countries have introduced comprehensive smoking bans in enclosed public and workplaces. This has, in countries where these bans are strictly enforced, significantly reduced exposure to SHS and its associated morbidity and mortality.^12^ However, for most non-smoking women and children, homes and cars remain important sources of SHS exposure. Additional measures are therefore required to protect non-smokers, particularly children, from SHS exposure. In Bangladesh, exposure to SHS has been recognised as a particularly serious threat to children's health given the high prevalence of smoking by male adults.^13^ However, smoking bans in public and workplaces are only partially implemented and little has been done to protect children from exposure to SHS at home. We carried out a recent household survey that revealed that most households (56%) have at least one smoker and that smoking indoors in front of children (40%) was a common practice.^14^

The evidence for the effectiveness of interventions to provide children a smoke-free environment at home is scarce. A recent Cochrane review concluded that, despite several studies on parental education and counselling programmes, their effectiveness in reducing children’s tobacco smoke exposure has not been clearly demonstrated.^15^ An alternative approach, for which there is also only very limited research, is to encourage children themselves to negotiate smoking restrictions in their households.^16^ We developed a school-based SFI and found through a feasibility trial in Dhaka, Bangladesh (24 schools, 781 children) that it was acceptable and successful in getting children to negotiate implementation of smoking restrictions at home (OR 3.9; 95% CI 2.0 to 7.5).^17^ Schools were also willing to participate and be randomised in a trial. Encouraged by these findings, we now wish to assess if the success of this intervention also translates to reduce SHS exposure in children and improves their health outcomes and academic achievements.

The principal question for our planned follow-on definitive trial is: What is the effectiveness and cost-effectiveness of a school-based SFI in reducing children's exposure to SHS (primary outcome), the frequency and severity of respiratory illnesses, healthcare contacts, school absenteeism, smoking uptake and in improving their lung function, quality of life and school performance?

Prior to conducting a definitive trial to answer the above question, we wish to first conduct a pilot trial that informs the parameters required for conducting the definitive trial.^18^ Our *specific research questions* for this pilot study are therefore to ascertain:

- What are the recruitment and attrition rates for schools (clusters) and children?
- What is the feasibility and acceptability of measuring the primary and secondary outcomes?
- What is the likely effect size and its variance in relation to the primary outcome measure?
- What is the intracluster correlation coefficient (ICC) among children for the trial outcomes?
- What is the fidelity of delivering SFI in schools?
- What are the costs associated with delivering SFI through schools?
- What would facilitate and hinder in scaling up SFI in schools and their curriculum?
- What is the frequency and nature of any adverse events (AE)?

## Methods and analysis

This is a two-arm pilot cluster randomised controlled trial (RCT) of SFI with an embedded preliminary health economic analysis and a qualitative evaluation. This is a 2-year study. We will recruit participants (schools and children) and start baseline assessments in the first 3 months. Between months 3 and 6, we will complete all baseline assessments, randomise schools and start delivering the intervention. We expect respective schools to have delivered the intervention by month 9, which means that all follow-ups will be completed between months 18 and 21. We will complete the analysis in the last 3 months and submit our final report at the end of 2 years.

### Study sites

The study will be conducted in Mirpur and Savar areas of Dhaka Division, Bangladesh. According to the 2011 census, these two sites geographically typify urban and peri-urban contexts, respectively, with a population of more than a million in each area. Mirpur is a typical densely populated urban area in Dhaka with the majority of its population dependent on non-agricultural livelihood and with access to amenities such as paved road, electricity, gas, water supply and drainage systems. On the other hand, Savar is a peri-urban area located 24 km northwest of Dhaka. The majority of its population is dependent on agriculture and industry located around Dhaka. These two areas have been chosen for their typical demographic and socioeconomic structures and existing links with the local communities, schools and health facilities.

### Study clusters (schools)

We will recruit 12 schools from the above two sites, six from each area. The key eligibility criteria are as below.

#### Inclusion criteria (schools)

We will include both public and private schools if they:

- Follow mainstream curricula approved by the educational authorities.
- Have year-5 classes, with >40 and <120 year-5 children (10–12 years old) per class.
- Have a ‘no-smoking’ policy and all participating year-5 teachers are self-reported non-smokers. It would be desirable to exclude those schools that have any year-5 teachers who smoke. However, given the difficulty in verifying smoking status, we will not make this a mandatory exclusion criterion.

#### Exclusion criteria (schools)

We will exclude schools if they:

- Have only primary school classes—these will be excluded due to the challenges of following up children as they move to a secondary school in year 6.
- Teach in English medium rather than in Bangla—such schools are in a minority in Bangladesh and usually cater for children from affluent backgrounds. There will be very few such schools in our proposed study sites.
- Have already received training on SFI in a previous project, unless the teachers who were trained have left the school.
- Are religious or faith-based schools not following the prescribed curricula.

#### Identifying and recruiting eligible clusters (schools)

We will survey the study sites and prepare a list of schools containing information on their class sizes in year 5, primary or secondary school status, public or private status, co-education or single sex schools and their medium (language) of teaching. Schools will be assessed for eligibility, using the criteria stated above. Once found eligible, these will be approached for recruitment. We acknowledge that some of the eligibility criteria can only be assessed after approaching schools and talking to the headmaster and year-5 teachers.

Those schools identified and found eligible on the basis of the available information will be sent a letter addressed to the head teacher, including brief information about the trial and inviting the school to take part in the trial. We will offer to meet head teachers face to face to provide verbal information and responses to their queries. We will also explain random allocation. Interested schools will be provided with a detailed information sheet.

Those who are not eligible or who meet the eligibility criteria but do not agree to participate after receiving the trial information will not be enrolled in the trial. However, their reasons for not meeting the eligibility criteria or declining to participate will be recorded.

Once recruited, we will endeavour to keep all schools on board and included in the study. If, for any reason, a school withdraws before randomisation, we will recruit a new school to replace the withdrawing school. However, if the withdrawal takes place after randomisation, we will not replace such a school and will include it in our analyses.

### Study participants (children)

We will recruit 360 year-5 (ie, at least 30 from each school) schoolchildren (10–12-years) after seeking their parents’ consent and their assent through schools. Being the oldest year in primary school years, we consider this year to be the optimal age group to understand the message and engage with their family members to implement smoking restrictions. The key eligibility criteria are as below.

#### Inclusion criteria (children)

We will include children if they are:

- Studying in year-5 in the participating school and their age range is between 10 and 12 years;
- Self-reported non-smokers.

#### Exclusion criteria (children)

We will exclude children if they have any of the following conditions/situations that the school is aware of:

- Physical or mental disabilities;
- Learning difficulties and/or special learning needs;
- Behavioural problems and/or conduct disorder;
- Serious medical condition which is either life-threatening or requires regular hospitalisation.

#### Identifying, recruiting and consenting eligible participants (children)

We will request schools to prepare a list of eligible children including all those who meet the inclusion criteria and excluding all those who fall into the exclusion criteria list. Once an eligibility list is prepared, we will give all schools the required number of trial information packs to proceed with the recruitment.

All children participating in this trial will be under 16 years and therefore parental/carer consent is required for them to take part. On the basis of our experience of conducting a previous study in schools in Bangladesh (approved by a national ethics committee) and advice received from a number of academics who work in schools in the UK, we will obtain parental consent on an opt-out basis as follows. The participating schools will send out the trial information packs to parents of all eligible children, containing an information sheet, and a parent/carer opt-out consent form. We will ask parents/carers to discuss the trial with their child/children. If either the parents or children are unwilling or unable to participate in the trial, we will ask the parents to indicate this by either sending us an opt-out consent form in a self-addressed prepaid envelope or calling/texting/emailing us on the contact details provided within the information pack. We will give parents/carers a minimum period of 7 days to indicate if they do not wish their child(ren) to take part in the study.

Moreover, at the time of recruitment, children will be of an age (10–12 years) where they are able to understand their potential involvement in research and can make an informed decision. Children will therefore be provided with an age appropriate information sheet, which will be given out to them at school. If children are unwilling, they can either let their teachers or parents know, as they feel appropriate. If parents indicate their disapproval for their child to take part in the study, this will supersede the child's assent to participate. Any child who has, or whose parents/carers have, declined to participate will be taken out from the list of eligible children by the school, and the final list will be handed over to the research team. All participating children will be given an enrolment number (including a code for school), which will be recorded on the final list of eligible children, printed on all enquiry tools and entered in the database.

Those children who do not meet the eligibility criteria, or those who meet the criteria but either their parents or they would not agree to participate after receiving the trial information, will not be enrolled in the trial. However, their reasons for not meeting the eligibility criteria or declining to participate will be recorded. This information will be kept completely anonymous.

A child can be withdrawn from participation at any time after enrolment or allocation. If a child is withdrawn from the intervention for any reason, their follow-up assessments and data collection will continue as per protocol unless the parents/carers or child specifically asks for their withdrawal from the study completely. However, if the child is withdrawn completely from the study, then no more data will be collected. They will still be included in the analysis and counted as lost to follow-up. The information already collected will be kept in the database unless the parents/carers or child specifically asks for their information to be removed.

### Cluster (schools) randomisation and allocation

Once the necessary baseline data have been logged into a database, the participating schools will be randomly allocated to each of the two arms, intervention and control, on an equal basis (ie, six schools in each arm). Each intervention school will be paired with a control school for follow-up purposes. We will use a restricted method of allocation called minimisation, which achieves balanced groups efficiently. For this, we will use three criteria, public/private status of school, boys to girls’ ratio, and mean number of children exposed to SHS at baseline.

### Intervention details

Once children are enrolled, schools will be randomised to receive either the SFI or treatment as usual. These treatment conditions are described as below.

#### Smoke free intervention

SFI is based on behaviour change techniques identified in the literature,^19^ and has been developed by a technical working group including schoolteachers, representatives of civil society organisations, public health practitioners, educational experts and behavioural scientists from Bangladesh and the UK. Once agreement was reached on the key messages, a range of educational materials, both in Bangla and English, were developed addressing different learning styles. The training materials for schoolteachers have been pre-piloted and revised according to the suggestions made by a user group and the technical working group. A manual has also been prepared to help schoolteachers in delivering SFI. It is envisaged that at least two year-5 teachers (one class teacher and one support teacher) will be trained in each school.

All participating children in the intervention arm will receive the SFI delivered by their teachers. Teachers will receive prior training in delivering the intervention. The intervention will consist of:

- Two 45 min sessions delivered over 2 days by schoolteachers. The duration of these sessions is consistent with regular school lessons. Each session consists of a range of educational activities including classroom presentations, quiz, interactive games, storytelling and role-play—vicarious learning techniques are utilised in many of these activities. The presentation, quiz and games are designed to increase pupils’ knowledge about SHS and related harms, and motivate them to follow three easy steps to make their homes smoke-free. The storybook and role-play focuses on enhancing children's negotiation skills, building their confidence within the Bangladeshi cultural context. While the storybook depicts the challenges of negotiating with elders, the role-play has hypothetical scenarios where children had an opportunity to practise and demonstrate how and when they can discuss and negotiate with elders to persuade them not to smoke inside homes.
- A set of four refresher sessions (15 min each) to reinforce key messages delivered in the initial sessions, to be delivered once a week over 6–7 weeks after the two initial sessions. During each session, the teacher reminds children of the key points of the main session by asking questions (5–7 min), and then encouraging students to share their experiences of whether they could initiate discussion at home, what challenges they faced, what is their plan to do next and what would be the best way to convince the elders (8–10 min). The length of these sessions is consistent with the duration of the school assembly.
- Children are given a promise form that contains pictorial and written messages on the hazards of SHS, a pictorial step-guide for families on how to make their homes smoke-free and a tear-off slip to make a commitment to impose smoking restrictions at home. Children take promise forms to their parents, show them the messages and negotiate with them to ‘sign-up’ to the SFI ‘promise’ form. One of the implications is that even if parents are non-smokers, they will not allow other smokers (residents and visitors) to smoke inside homes. In addition to delivering the intervention, teachers will also be trained to support children in this process.

#### Treatment as usual

Schools in the control arm will receive the intervention at the completion of the trial.

### Outcomes assessment

Specific outcomes for this pilot trial include: (1) number of clusters (schools) and the size of each cluster (children) for the main trial; (2) recruitment and attrition rates for clusters and participants; (3) length of time required to reach participant recruitment saturation for each cluster; (4) descriptive data on characteristics of participating clusters and participants; (5) reasons for ineligibility of clusters and participants; (6) reasons for non-participation/non-consent of clusters and participants; (7) estimate of the effect size of the primary outcome in a definitive trial (salivary cotinine); (8) calculation of the ICC for the trial outcomes in order to inform the sample size required for the main trial; (9) estimate of contamination between clusters; (10) the costs associated with delivering SFI; (11) fidelity of the intervention and (12) the time and resources required in measuring all outcomes, including the response rate to obtaining saliva samples and the extent and type of missing data with reasons. We will assess and report on these outcomes as part of our data collection and analysis plan.

The outcomes for the definitive trial will also be measured before and after the intervention in each of the study's arms. We first describe these outcomes and then the process and schedule of assessing these (table 1).

**Table 1:** Table and schedule of assessments within CLASS II trial

	Study period						
	Enrolment	Allocation	Postallocation				Close-out
Time points	Baseline—t_0_	0	Month 1—t_1_	Month 2—t_2_	Month 6—t_3_	Month 12—t_4_	Month 15—t_x_
*Enrolment*							
Eligibility screen	X						
Parental consent	X						
Children's consent	X						
Allocation		X					
*Interventions*							
Smoke-free intervention			fx1				
Controls			fx2				
*Assessments*							
Sociodemographic and medical history							
Personal details	X						
Household details	X						
Medical history	X						
Smoking-related behaviours							
Smoking restrictions	X			X	X	X	
Attitudes towards smoking	X			X	X	X	
Health service use	X			X	X	X	
Quality of life	X			X	X	X	
Exposure to SHS—salivary cotinine	X			X			
Lung health	X			X			
Respiratory symptoms diary			X	X	X	X	
Lung functions—spirometry	X			X	X	X	
Academic assessment							
Academic performance questionnaire	X			X	X	X	
School absenteeism report	X			X	X	X	

CLASS, Children Learning About Secondhand Smoke; SHS, secondhand smoke.

### Primary outcome

We will assess children's exposure to SHS by measuring their salivary cotinine levels. Salivary cotinine concentration, a sensitive biochemical marker, is strongly associated with the exposure to SHS at home. It has a half-life of 20 h. It is a widely recognised method for detecting both active and passive smoking and has been used in several surveys and studies. It involves keeping a sterile swab in the mouth for a few seconds, allowing it to absorb saliva and then transferring it to a sterile plastic container. The samples are subsequently analysed and a gas-liquid chromatography technique can detect cotinine levels as low as 0.1 ng/mL.

Once children are enrolled in the study, saliva samples will be obtained from all participating children at baseline and also at 2-month post-intervention. Samples can be stored for a period of 2 weeks before being transported to a specialist laboratory (ABS Labs) in the UK for these to be analysed using a gas-liquid chromatography technique. Samples will be sent in two batches at baseline and two at the first follow-up. Samples will not contain any participant identifiable information and will only have the trial enrolment number. Their reports will be sent back to the central research office in Dhaka where these will be entered in the database.

### Secondary outcomes

These will include the below.

#### Frequency and severity of respiratory symptoms

Participating children will keep diaries and record respiratory symptoms on a nominal severity scale from 0 to 3 as used by Chauhan *et al*.^20^ For each item, ‘0’ representing the absence of a symptom, 1 representing mild, 2 representing moderate and 3 representing the greatest severity level. The upper respiratory tract symptoms will include having a runny nose or sneezing, blocked or stuffy nose, sore throat or hoarse voice, headaches or face aches, aches or pains elsewhere, and feeling chill, fever or shivers. The lower respiratory symptoms will include cough on waking, wheeze on waking, cough during the day, wheeze during the day, shortness of breath during the day, night cough and wheeze or shortness of breath during the night. Scores will be recorded daily and added up to give daily upper and lower respiratory-tract scores, respectively.

Three hundred and sixty diaries to record respiratory symptoms will be printed and given to all participating children. These diaries will have three sections. Section 1 will record symptoms from the day intervention is delivered until the end of month 2; section 2 from the start of month 3 until the end of month 6; and section 3 from the start of month 7 until the end of month 12. At each follow-up, one section will be taken out of the diary by the researchers and data will be entered in the database. All children will provide data on section 1. Only those children whose cotinine levels are indicative of passive smoking at baseline will provide data on sections 2 and 3. Children will be told whether to stop or keep collecting information in their diaries in a letter.

#### Lung functions

We will measure lung functions on all participating children at baseline and on children with positive salivary cotinine at months 2, 6 and 12. For this, we will use a spirometer as per British Thoracic Society guidelines.^21^ This will involve measuring forced vital capacity, forced expiratory volume in the first second (FEV1) and peak expiratory flow using a handheld Micro1 spirometer. We will first record the height of all participating children at baseline. We will enter details of the patient's sex, ethnic group, age and height into the spirometer. We will then ask each child to blow into a disposable mouthpiece attached to the spirometer at least three times as per guidelines. The best of the three readings will be taken. The spirometer will also calculate the percentage of the predicted normal values (using Asian reference values) as they have the reference data already programmed into them. The spirometer will print out a lung function test report for each participant, which will be attached to the participant baseline and follow-up questionnaire, respectively. Spirometers will be cleaned according to the manufacturer's guidelines and their accuracy checked regularly.

#### Smoking-related behaviours

We will ask the children to self-report levels of smoking restrictions and social visibility of smoking at home through a questionnaire. We will assess smoking restrictions using the following questions: (1) ‘Where do people smoke in your house who live with you? (Anywhere inside the house, in some rooms, only in one room, or only outside the house)’; (2) ‘Where do smokers who visit your house smoke? (Anywhere inside the house, in some rooms, only in one room, or only outside the house)’. We define ‘open space outside house’ as those spaces, which are still within the house premises but not covered by a ceiling, such as the veranda, balcony, yard, garden, lawn, patio and open roof. Social visibility will be assessed by the following questions; (1) ‘Do people who live with you smoke in front of children?’ (2) ‘Are people who visit your house allowed to smoke in front of children?’ For each outcome, the response categories across the two questions will be combined to form a composite variable (index) for analysis purposes.

Using the same questionnaire, we will also assess children's self-reported attitude towards smoking and intention to start smoking. We will use a five-point smoking uptake scale^22^ to categorise children as non-susceptible non-smokers, susceptible non-smokers, early experimenters, advanced experimenters and established smokers.

The above assessments will be carried out both at baseline and at all follow-up time points.

#### Health service use

We will use a health service utilisation questionnaire previously used in the Muslim communities learning about secondhand smoke (MCLASS) trial,^23^ to collect the number and type of contacts with doctors/nurses, hospital admissions, pharmacy visits and antibiotic prescriptions. This information will be part of the baseline questionnaire but will also be assessed at all follow-up time points.

#### Quality of life

Quality of life (generic) will be assessed using a short quality of life questionnaire for children EQ-5DY.^24^ The questions will be included in the baseline and follow-up questionnaires.

#### Other confounding variables

At baseline, we will also ask children to report on some of the basic sociodemographic details on the questionnaire. These will include age, gender, household amenities, family structure, cohabiting smokers—including parents, pet ownership, overcrowding—number of rooms and residents, built environment, neighbourhood, presence of mould/moisture in the child's home, and use of gas for cooking or gas/kerosene/oil heater. Furthermore, we will include information on children's medical history (particularly asthma and chest infections) and use of any regular medications.

#### Absenteeism and academic performance

Each participating school will be asked to provide a report on the academic performance of participating children using the Academic Performance Questionnaire (APQ).^25^ This is a 10-item questionnaire to be completed by teachers. Using four-point and five-point ordinal scales, it measures a child's performance in reading, mathematics, writing and homework. This questionnaire will be completed at baseline and at all follow-ups. In addition, schools will also be requested to provide a record of child's absenteeism from school including the number of days missed every month in between two assessments.

#### Fidelity index

The research team will use a fidelity index, mapped onto the behaviour change techniques that underpin SFI, to assess intervention adherence. This will be in the form of a checklist, which will be used to monitor the delivery of SFI sessions. One of the members of the research team will attend all SFI sessions and using the above checklist will score fidelity to SFI.

### Data collection

Prior to randomisation, a baseline assessment will include a classroom-administered questionnaire (including EQ-5DY, health service use and smoking behaviour) to be completed by participating children, APQ and school absenteeism form to be completed by school teachers, lung function assessment and saliva sample collection by the research team for each child (table 1). Each child will also receive a respiratory symptoms diary with instructions on how to use it. Follow-up assessment will take place at 2, 6 and 12 months post-allocation involving only those children whose cotinine levels were indicative of SHS exposure at baseline. All assessments carried out at baseline will be repeated at the follow-up assessment except cotinine levels which will only be assessed at month 2.

### Qualitative evaluation

Teachers and head teachers (2 and 1 per school; 12 and 6 in total, respectively) from the six intervention schools will be interviewed once the SFI has been delivered. Semistructured interviews will explore the barriers and facilitators to delivering the different components of the SFI, including training and resource needs. Interviews will be conducted using a topic guide to ensure consistency, with flexibility to allow participants to generate naturalistic data on what they see as important. Where possible, the interviews will be conducted face to face at school. If this is not possible, then telephone interviews will be done. With the participants’ permission, interviews will be audio recorded digitally and transcribed verbatim with anonymisation of all personal data.

### Data management and archiving

Data will be initially collected in the form of a paper-based questionnaire. Containing no participants’ identifiable information, these will be collected by the research team and kept in a locked cupboard separate from the consent forms. Once a week, data will be entered in the trial database, which will be kept at a secure server. The paper copies will continue to be kept in a securely locked cupboard.

In line with the Data Protection Act and the Research Governance Framework for Health and Social Care Research, at the end of the trial, data will be securely archived by the University of York for a minimum of 5 years. Following authorisation from the sponsor, arrangements for confidential destruction will then be made. If an individual withdraws consent for their data to be used, it will be confidentially destroyed.

### AEs procedures

We are expecting a minimal number of adverse and no serious AEs (SAEs) during the study. SFI is an educational intervention and has been very well received in our previous studies^17^ without leading to any directly related AEs. Nevertheless, there will be a vigilant surveillance system in place for AEs occurring during the course of the trial with particular emphasis on identifying, recording, reporting and managing any SAEs. We will sensitise schoolteachers to look for signs of any AEs resulting from the interactions between children and their parents. We will also encourage children and parents to report any related adversities.

#### Definitions

##### Adverse event

An AE is any untoward clinical event in a trial participant, which may or may not be related to the study intervention. The clinical event could be an unfavourable and unintended symptom, sign, medical condition, abnormality or disability that has appeared or worsened during the course of the trial, regardless of a causal relationship to the study intervention.

##### Serious adverse event

A SAE is any clinical occurrence that:

- Results in death of the participant;
- Is life-threatening, defined as an event in which the participant is at risk of death during the event. This does not refer to incidents that hypothetically might have led to death if the event worsened;
- Results in hospitalisation or prolongation of hospital stay;
- Results in persistent and/or significant disability and/or incapacity;
- Birth defect or congenital anomaly;
- Any medical condition that may not be life-threatening, disabling or resulting in hospitalisation but requiring medical or surgical intervention to prevent any of the above outcomes.

Please note that any planned surgery or medical procedure will not be considered as an SAE.

#### Detecting, recording and reporting of AEs and SAEs

In the event of any AE reported by the child, their parents/carers or schoolteachers, the research assistant will complete an AE form, which will include medical diagnosis, if relevant and available. The research assistant will also call the trial manager on the same day providing a verbal report of the event. The trial manager will ensure that the event is classified appropriately after receiving the verbal report. All AEs will be reported to the principal investigator, Bangladesh (RH) within 3 days of detection. AE data will be collated and reported to the trial sponsors and National Bioethics Committee at six monthly intervals. These must also be reported to the Study Operational Committee (SOC) and the Independent Trial Steering Committee (ITSC) at their regular meeting. All AEs that have the potential to develop into SAEs will be followed to resolution or stabilisation and reported as SAEs if they become serious. All SAEs must be reported to the principal investigator within 24 h of detection and should also be reported to the trial sponsors and National Bioethics Committee within three working days. All serious events must also be reported to all study investigators and the chair of the ITSC within 3 days. The chief investigator will have the overall responsibility to ensure that all AEs are reported according to the above protocol.

#### Evaluation of AEs and SAEs

In addition to assessing seriousness, the trial manager will assess all AEs for causality, severity and expectedness.

##### Assessment of causality and relatedness

This will be done in consultation with the principal investigator and the event will be classified as follows.

*Unrelated*: When the event is considered not related to the study intervention.

*Possibly*: When an association of the event with the study treatment cannot be ruled out.

*Probably*: When temporal association and an absence of any other explanation suggest that the event could be related to the study intervention.

*Definitely*: Evidence suggests that the study intervention is the most likely cause of the event.

*Assessment of severity*: The trial manager will make the following assessment based on severity, which should not be confused with seriousness (a statutory definition) differentiating between AEs and SAEs.

*Mild*: Those events that cause minimal discomfort, are easily tolerated and do not interfere with routine life activities.

*Moderate*: Those events that cause moderate discomfort and do interfere with routine life activities.

*Severe*: Those events that cause much discomfort and lead participants to stop their routine life activities.

##### Assessment of expectedness

If the event is judged to be an adverse reaction, serious or otherwise, it must be judged on expectedness based on what is already known about the intervention under study.

#### Follow-up procedures

These events will be followed up until resolution or returning to a stable medical state. We will not expect any events to be relevant to the trial that occur after the completion of follow-up, and therefore no active surveillance will continue beyond trial completion. Nevertheless, any event reported to the trial manager will be recorded and kept in the records along with other trial data.

### Statistical considerations

#### Sample size

This pilot trial will inform sample size considerations for a definitive trial. Therefore, no formal sample size calculations have been undertaken. However, with the number of clusters (and cluster size) we seek to enrol, we should be able to estimate recruitment and attrition rates, effect size and ICC. For our primary outcome, the pilot trial will provide us with a more accurate effect size and SD values. If the effect of intervention is similar to that observed in our feasibility study and implementing smoking restrictions at home imply a reduction in SHS exposure, we should expect 40–50% reduction in children's exposure to SHS. The ICC observed in our feasibility trial was 0.18, but it was estimated for a proximal outcome. Therefore, in this study, we will be able to determine a more accurate estimate of ICC based on a distal outcome. Assuming that at least 360 children (and their parents/carers) agree to participate from each school, we expect at least 120 children with a positive cotinine test as baseline. On the basis of the feasibility trial, we expect no schools to drop out from the trial and the children's attrition rate to be <20%. According to this, we estimate that we will be able to retain at least 96 children by the end of the trial. However, actual attrition rates will be found to make adjustment to the sample size.

#### Statistical analysis

We will conduct a preliminary analysis summarising: participant (individual and cluster) characteristics; attrition rates; likely estimates of effect and ICC for the outcomes. This will also include a comparison between participants with detectable and zero levels of cotinine at baseline. Although determining differences in the outcomes between the two arms is not the purpose of this study, we will summarise outcomes at both cluster and individual levels using an intention-to-treat principle and estimate 95% CIs for any differences. All analyses will be conducted using SAS 9.4 (Institute Inc, Cary, North Carolina, USA).

### Qualitative evaluation

Interview data will be analysed using thematic analysis,^26^ which is a method of ‘identifying, analysing and reporting patterns (themes) within the data’. This is a useful approach for producing qualitative analyses suited to informing programme evaluation and development. The focus of the analysis will be to identify key issues in delivering the SFI that may inform the process of ‘scaling up’ the intervention for the definitive trial. Six phases of thematic analysis will be followed: familiarisation, generating initial codes, searching for themes (prior—informed by study objectives as well as emergent), reviewing themes, defining and naming themes, writing the final report. Negative cases will be actively sought throughout the analysis and emerging ideas and themes modified in response.^27^ NVivo will be used to manage the data.

### Health economics

In the pilot phase of this trial, we will assess the feasibility and acceptability of undertaking a full cost-effectiveness analysis. The first stage will estimate the cost of delivering the intervention. Intervention costs will include the time and resources needed to deliver the SFI intervention. We will also pilot a service use questionnaire to record utilisation of healthcare resources. These include doctor and hospital visits for the treatment of childhood conditions related to the inhalation of passive smoke and also medications dispensed for such illnesses. We will audit and analyse the returned data for completeness and revise questionnaires where appropriate to improve data capture in a full trial.

The purpose of the pilot phase is to inform a cost-effectiveness analysis in a full trial with regard to intervention cost and data collection. In a full trial, we will conduct an incremental cost-effectiveness analysis of the smoke-free homes intervention over and above the control. Total costs include the costs of the intervention (SFI) as outlined above and wider healthcare costs such as doctor and hospital visits for childhood illness related to the inhalation of passive smoke (asthma, wheezing, middle ear infections, respiratory tract infections and meningitis). We will also record and calculate the costs of medications related to these illnesses, which are dispensed. In the full trial analysis, the quantities of resource use (contacts) are multiplied by local unit costs to derive an individual cost profile.

The main outcome for the health economic analysis of the full trial will be changes in quality adjusted life years (QALYs), which will be computed from the EQ-5DY questionnaire^24^ completed by trial participants. In a full trial, we propose a within-trial cost-effectiveness analysis combining the costs of the intervention and wider treatment with QALY gains to derive an incremental cost per QALY. The cost per QALY will inform the value for money afforded by investment in the SFI intervention. We will perform sensitivity analysis to investigate the robustness of the results. We will also explore potential differences in cost-effectiveness between centres within the trial using multilevel modelling techniques.

### Study organisational structures

The study will be managed and steered by a SOC and an ITSC. The SOC will consist of all study investigators and will be responsible for the delivery of the project. It will meet on a bimonthly basis over Skype/teleconference and will review trial progress, respond to any concerns raised by the trial manager and the principal investigator, propose remedial actions and detect any forthcoming problems. The ITSC will be set up and will include an independent chair, at least two other independent members, a chief investigator, a research fellow, a trial manager, a trial statistician and the principal investigator. It is likely to meet every 6 months but will decide on the frequency of meetings. The ITSC will oversee the trial and ensure that the trial is conducted according to the protocol and within the underlying ethical framework. Members will also provide advice outside these meetings according to their area of expertise at key stages via email, phone or, if needed, face to face.

### Data monitoring

Data will be monitored for quality and completeness first by the trial manager and then by the principal investigator (Bangladesh) using verification, validation and checking processes. Once data are entered into the database, all entries for the primary outcomes and a random sample of 10% entries for other variables will be double checked by the trial manager. In addition, all paper-based forms will be double-checked and validated against the original forms.

### Protocol amendment

All amendments to the protocol will be first discussed with the chief investigator and then submitted to the Bioethics Committee for formal approval. A judgement will be made on the nature of amendment, that is, major or minor, as per guidance from the Bioethics Committee. All minor amendments will be implemented once notified to the Bioethics Committee and all major amendments will be implemented once approved by the Bioethics Committee.

### Protocol violations and deviations

The research team will not deviate from the protocol without agreement with the chief investigator and securing an agreement with the Bioethics Committee and SOC except in situations where a deviation is necessary to remove an immediate hazard to the participants. Any such deviations (both nature and reason) should be recorded in the AE form and, if necessary, an amendment to the protocol must be secured through a formal process.

### Quality assurance

The study will be conducted in accordance with current MRC Good Clinical Practice guidelines and the NHS Research Governance Framework.^28^ Administrative approval will be sought from each participating school. The study will be subjected to all research management and governance procedures in place at the University of York including the requirement for audit.

## Ethics and dissemination

The trial will be conducted to protect the human rights and dignity of the participant as reflected in the 1996 version of the Helsinki Declaration. Participants will not receive any financial inducement to participate in the trial. In order to protect the trial participants, the following provisions will be made/upheld; the trial has been designed to minimise the burden of participants and any foreseeable risk in relation to the intervention involved; the explicit wishes of the participant will be respected including the right to withdraw from the trial at any time; the interest of the participant will prevail over those of science and society; provision will be made for indemnity by the investigator and sponsor.

We have received ethical approval from the University of York and the Bioethics Committee of Bangladesh Medical Research Council.

Some of the ethical issues considered are as follows: (1) children's participation raises issues around competence, vulnerability and powerlessness. In this study, children's wishes and their welfare will take precedence over research requirements. Research burden will be kept to a minimum. (2) Children and their families will not be reimbursed financially; however, small incentives in the form of school stationery will be offered. (3) On the basis of our feasibility work, it is highly unlikely that the children will face any adverse reaction from their families. Obtaining saliva sample for cotinine is also not harmful to children, neither it could disclose presence of any medical condition. (4) All participants’ data will be kept confidential and in password-protected servers.

### Confidentiality

All information collected during the course of the study will be kept strictly confidential. Information will be held securely on paper and electronically at the central research office. The researchers will also comply with all aspects of the 1998 Data Protection Act and operationally this will include:

- Consent from participants to record personal details including name, date of birth and address.
- Appropriate storage, restricted access and disposal arrangements for participant's personal details.
- Identifiable information will be collected on the consent form when he or she is consented into the trial, but all other data transferred to or from the central research office will be coded with a trial enrolment number and will not include participants’ identifiers.

If a participant withdraws consent from further trial participation (please note that this would be different from withdrawing from the study intervention), no further assessments will be conducted but their data already collected will remain on file and be included in the final study analysis (unless the participant has specifically requested for their data to be destroyed).

### Dissemination plans

This is a proposal for a pilot cluster RCT of SFI school-based intervention, and therefore its findings will inform a future definitive trial rather than the effectiveness and cost-effectiveness of the intervention. Nevertheless, the potential impact of this study and the subsequent research is likely to be high as it addresses a key priority in children's health, uses an innovative behavioural approach, employs a robust study design, engages with primary schools and attempts to measure a variety of health and educational outcomes. Our proposed pilot and the subsequent definitive trial could lead to:

- A wider use of SFI or similar approaches to address SHS exposure. If shown to be successful, this could lead to improving children's physical health and academic performance. It will also contribute towards shifting social norms of smoking in the house and in front of children, which could encourage smokers to consider quitting. If it helps in changing children's own attitudes towards smoking, SFI could become a successful preventative measure.
- If shown to be successful, the behavioural approaches and settings used in our study could also be used to tackle other unhealthy behaviours such as poor diet and physical inactivity. Children's health behaviours are often determined by parenteral and sibling's attitudes and behaviours. Our proposed approach lends itself nicely to address other health behaviours and can result in improving family health in ways that go beyond what is proposed here.
- Our proposed methods and measures can help researchers from a range of disciplines. Such methods and measures are rarely used in low-income settings causing a dearth of experience and related data. Our study will provide both experience and useful data in a setting and population group where both are scanty. Other researchers would benefit from this and can use parameters generated from this research to design and conduct their own studies.

The following is a list of academic and non-academic audience for this study with a set of activities that will be undertaken to engage with them to maximise its impact.

#### Academic audience

- Public health researchers interested in generating evidence for school-based interventions aimed at improving children's health.
- Behavioural scientists interested in building an evidence base for behaviour change techniques.
- Trialists with an interest in school-based trials in low-income settings.
- Clinicians who would wish to use lung health measures in studies where the end points are similar to ours.
- Education researchers who are interested in studying the relationship between health, school absenteeism and academic performance using standardised instruments.
- Health service researchers interested in children's health and their use of health service.
- Researchers in tobacco control interested in measuring SHS exposure in children.
- Health economists who wish to carry out economic evaluation of school-based interventions.
- Students of health, medicine and social care will benefit as learning from this research will be incorporated within the relevant curriculum.

##### Ways of engagement

- We will publish the results of the trial in a high-impact peer-reviewed journal
- We will also publish the results of the qualitative component of the research in a journal specialising in smoking issues (eg, *Tobacco Control*) or general public health issues (eg, *BMC Public Health*)
- We will publish our pilot trial protocol in an open access journal. In addition, we will also present our findings in an international public health conference and an international tobacco control conference
- We will also publish an abstract of our findings on our website
- We will also present our work to regional research meetings
- We will also work with our network of other researchers (World Universities Network (WUN) Education group) and jointly submit a systematic review of smoking prevention and cessation interventions using school-based approaches
- We will incorporate the learning from this project into health promotion lectures for Master students of Public Health.

#### Non-academic audiences

There are several non-academic audiences that we wish to reach. These include the following:

- The WHO Tobacco Free Initiative is part of the Non-communicable Diseases and Mental Health cluster at the WHO headquarters in Geneva, Switzerland;
- The Tobacco section of the International Union Against TB and Lung Diseases;
- The National Tobacco Control Cell (NTCC), which is the functional arm of the Ministry of Health and Family Welfare for tobacco control activities in Bangladesh;
- Bangladesh Anti-Tobacco Alliance (BATA), which includes Non-governmental organisations (NGO) representatives who actively work in tobacco control activities in Bangladesh;
- Bangladesh's National Curriculum & Textbook Board;
- Individual schools;
- Local leaders and community representatives;
- Policymakers, ward commissioners and the Upazila Chairman who have an interest and also the responsibilities to address health inequalities and the health needs of communities;
- Health and social care professionals.

##### Ways of engagement

- We will establish a study Technical Working Group (TWG). This will comprise researchers, policymakers and representatives of the Tobacco Control Cell, BATA, schoolteachers and community members
- At all stages of the project, we will carry out meetings/workshops in collaboration with the National Tobacco Control Cell. We will invite schools, other NGO representatives, community leaders and community members in these workshops. The objective will be to raise awareness about the smoking related issues and explore ways of engaging with schools in health promoting and research activities
- We will produce a written report and a policy brief of research process and results
- We will also disseminate our research findings in national and international conferences/seminars
- We will also use opportunities like World No Tobacco Day, World TB Day to publicise our work by distributing leaflets and project briefs and doing community talks

### Authorship eligibility guidelines

The success of the study depends on the collaboration of all participants. For this reason, credit for the main results will be given to all those who have collaborated through authorship and contribution.

Uniform requirements for authorship for manuscripts submitted to medical journals will guide authorship decisions. These state that authorship credit should be based only on substantial contribution to:

1. Conception and design, or acquisition of data, or analysis and interpretation of data;
2. Drafting the article or revising it critically for important intellectual content;
3. Final approval of the version to be published;
4. That all these conditions must be met.

Seen in this light, the chief investigator, co-investigators, principal investigator (Bangladesh) and the trial manager will be named as authors in any publication if they satisfy the above criteria. In addition, all collaborators will be listed as contributors for the main trial publication, giving details of roles in planning, conducting and reporting the trial.
